# Parents’ experiences of childhood abuse and neglect are differentially associated with behavioral and autonomic responses to their offspring

**DOI:** 10.1002/dev.21822

**Published:** 2019-02-06

**Authors:** Renate S. M. Buisman, Marian J. Bakermans‐Kranenburg, Katharina Pittner, Laura H. C. G. Compier‐de Block, Lisa J. M. van den Berg, Marinus H. van IJzendoorn, Marieke S. Tollenaar, Bernet M. Elzinga, Jolanda Lindenberg, Lenneke R. A. Alink

**Affiliations:** ^1^ Centre for Forensic Family and Youth Care Studies Leiden University Leiden The Netherlands; ^2^ Leiden Institute for Brain and Cognition (LIBC) Leiden University Leiden The Netherlands; ^3^ Clinical Child & Family Studies VU University Amsterdam Amsterdam The Netherlands; ^4^ Primary Care Unit, School of Clinical Medicine University of Cambridge Cambridge UK; ^5^ Institute of Psychology, Clinical Psychology Unit Leiden University Leiden The Netherlands; ^6^ Department of Psychology, Education and Child Studies Erasmus University Rotterdam Rotterdam The Netherlands; ^7^ Leyden Academy on Vitality and Ageing Leiden The Netherlands

**Keywords:** childhood maltreatment, parenting behavior, physiological reactivity, pre‐ejection period, respiratory sinus arrhythmia

## Abstract

Although childhood maltreatment has been shown to compromise adaptive parental behavior, little is known what happens in terms of physiological regulation when parents with a history of childhood maltreatment interact with their offspring. Using a sample of 229 parents (131 women), the present study examined whether childhood maltreatment experiences are associated with parents’ behavioral and autonomic responses while resolving conflict with their offspring. Self‐reported experienced child maltreatment was measured using a questionnaire assessing abuse and neglect. Parents (*M*_age_ = 52.7 years, range_age_ = 26.6–88.4 years) and their offspring (*M*_age_ = 24.6 years, range_age_ = 7.5–65.6 years) participated in a videotaped parent–offspring conflict interaction task. Parental warmth, negativity, and emotional support were coded. In addition, their pre‐ejection period and respiratory sinus arrhythmia were measured as indicators of underlying sympathetic and parasympathetic nervous system reactivity, respectively. Findings demonstrated that experiences of abuse and neglect were associated with behavioral and physiological responses in different ways. Separating these two types of maltreatment in research and in clinical practice might be important.

## 1 INTRODUCTION

Experiencing maltreatment during childhood imposes significant stress on children as for the child the caregiver is both the source of potential comfort and the source of threat and distress (Main & Solomon, 1990). Not surprisingly, childhood maltreatment experiences are associated with a wide range of negative consequences such as emotional and behavioral dysregulation in childhood (Cicchetti & Toth, 2005) and subsequent adverse mental health outcomes in adulthood (Jaffee, 2017). They have also been shown to compromise adults’ interpersonal functioning including their parenting behavior (e.g., Pears & Capaldi, 2001). However, little is known about what happens in terms of physiological responses and regulation when parents with a history of childhood maltreatment interact with their offspring. Although physiological regulation and reactivity have been shown to be affected by childhood maltreatment experiences (e.g., Buisman et al., 2018; Casanova, Domanic, McCanne, & Milner, 1994) and are considered to underlie parenting quality (e.g., Bridges, 2008), empirical research on the link between parents’ childhood maltreatment experiences and their physiological reactivity during real‐time parent–offspring interactions is lacking. Furthermore, it may be important to distinguish between experiences of abuse versus neglect when examining the consequences of child maltreatment. Child neglect implies an act of *omission* and is related to the failure to meet the child's physical and emotional needs, whereas child abuse implies acts of *commission* which can be verbal or physical. Both abuse and neglect have been linked to maladjustment, yet it is still unclear whether abuse and neglect differentially impact behavioral and physiological systems (Gunnar & Quevedo, 2007). Accordingly, in an effort to expand the knowledge regarding childhood maltreatment and subsequent parenting behavior, the present research examines how parents’ childhood experiences of abuse and neglect are associated with their physiological reactivity and parenting behavior—specifically warmth, negativity, and emotional support, during a parent–offspring conflict interaction task.

### 1.1 Childhood maltreatment history and parenting behavior: theoretical perspectives

Several perspectives may be useful to help explain the association between childhood maltreatment and later maladaptive parenting behavior. First, developmental psychopathology suggests that early childhood trauma impacts the quality of caregiving behaviors through stress or trauma‐related symptoms that undermine caregiving capacities. Childhood maltreatment has been associated with heightened attentional and affective responses to fearful and angry faces, deficits in emotion recognition, and reduced responsiveness to reward in children and adults (Jaffee, 2017). These distorted affective responses may compromise parents’ capacity to respond in an appropriate way to their children's emotional needs and behaviors.

Attachment theory has also proposed explanations for linkages between childhood maltreatment and subsequent parenting difficulties. According to Bowlby (1969), children develop “internal working models” (i.e., mental representations) of the self and others through repeated interactions with their primary caregiver(s), from which they then interpret and experience other relationships, including the relationship with their own offspring. In line with this proposition, empirical research has shown that adults who experienced childhood maltreatment were more likely to form insecure working models (Raby, Labella, Martin, Carlson, & Roisman, 2017), and insecure working models in turn have been associated with maladaptive caregiving (Dykas & Cassidy, 2011; Reijman et al., 2017).

According to social learning theory, behavior is learned in large part through observation, imitation, and reinforcement (Bandura, 1973). It postulates that violence is learned through role models provided by the family either directly or indirectly (i.e., witnessing violence), is reinforced in childhood, and continues in adulthood as a coping response to stress or as a method of conflict resolution. Support for this theory comes from studies that showed that the experience of violence in childhood is associated with general patterns of violent behavior (e.g., Widom, 1989), as well as later violence in close relationships (e.g., Ehrensaft et al., 2003; Pears & Capaldi, 2001).

Finally, at a neurobiological level, chronic childhood maltreatment is thought to increase allostatic load (Cicchetti & Toth, 2005). According to McEwen's (1998) theory of allostatic load, the autonomic nervous system (ANS) attempts to maintain stability through change during stressful conditions in order to maximize survival, a process called allostasis. When the system is exposed to repeated or chronic stress, including child maltreatment, physiological responses may become dysregulated, a process referred to as allostatic load. Two common patterns of allostatic load are *hyper*‐ and *hypo*‐arousal. A *hyper*‐arousal response pattern is characterized by heightened physiological activity and dampened physiological recovery in response to a stressful event. A *hypo*‐arousal response pattern is denoted by dampened physiological activation to environmental stress and challenge. Both patterns of allostatic load are thought to diminish parental capacity to call upon effective parenting behaviors and strategies (Bridges, 2008; Sturge‐Apple, Skibo, Rogosch, Ignjatovic, & Heinzelman, 2011).

### 1.2 Childhood maltreatment history and parenting behavior: empirical research

A number of empirical studies have investigated the association between childhood maltreatment and later parenting. Studies using self‐report measures of parenting behavior revealed that child maltreatment is associated with maladaptive parenting outcomes including lower perceived parenting competence, more parenting stress, more role reversal, decreased responsivity, more harsh physical discipline, and more abusive and neglectful parenting behaviors (Alexander, Teti, & Anderson, 2000; Banyard, 1997; Bennett, Sullivan, & Lewis, 2006; Bert, Guner, & Lanzi, 2009; Dilillo & Damashek, 2003; Heyman & Smith Slep, 2002; Lang, Gartstein, Rodgers, & Lebeck, 2010). However, it is questionable whether parents’ subjective estimation of their parental behavior reflects their actual parental functioning, because their reports may be biased by their ability to accurately reflect on and describe their interpersonal behavior (Bailey, Moran, & Pederson, 2007; Chiesa & Fonagy, 2014). Indeed, convergence between self‐reported and observed parental interactive behavior with their offspring is typically low, particularly in mothers from high‐social‐risk populations (Bailey, DeOliveira, Wolfe, Evans, & Hartwick, 2012; Bennett et al., 2006; Fitzgerald, Shipman, Jackson, McMahon, & Hanley, 2005). As opposed to self‐reports of parenting, observational measures of parenting are considered to be less influenced by bias (Gardner, 2000) and have been shown to be stronger and more consistent predictors of offspring outcomes (Zaslow et al., 2006).

In their literature review, Vaillancourt, Pawlby, and Fearon (2017) identified fourteen studies on the association between maternal experiences of childhood physical and sexual abuse and observed parent–infant interactions. Of these fourteen studies, ten showed that childhood abuse experiences were, directly or indirectly, associated with maternal interactive behavior, including hostile, intrusive, and inconsistent behavior toward infants. The available research on childhood maltreatment history and observed parent–offspring interactions with children during later developmental stages (toddlerhood and middle childhood) revealed that mothers’ history of childhood maltreatment was associated with less sensitive, more hostile, and more self‐focused behavior (Bailey et al., 2007; Burkett, 1991; Pasalich, Cyr, Zheng, McMahon, & Spieker, 2016; Rahma, Alsarhi, Prevoo, Alink, & Mesman, 2018). Studies on childhood maltreatment and observed parental interactions with offspring beyond middle childhood are—to our knowledge—lacking, even though the parent–offspring relationship constitutes one of the most long‐lasting social relationships and continues to be of importance in the lives of most adults (e.g., Trommsdorff, 2006). In addition, the majority of studies did not include any fathers, whereas fathers’ involvement in childcare has continuously increased in many western countries the past few decades (Jones & Mosher, 2013; World Health Organization, 2007). Finally, among the set of studies with observational measures, most investigated the impact of physical abuse, leaving the question open whether the effects also hold true for other types of maltreatment such as emotional or physical neglect. In the current study, we therefore examined the impact of childhood abuse *and* neglect in maternal as well as paternal interactions with offspring spanning a wide age range.

### 1.3 Continuity in parent–offspring interactions

While transitions from childhood to adolescence and from adolescence to adulthood may generate changes in parent–offspring relationships (e.g., Buhl, 2007; McGue, Elkins, Walden, & Iacono, 2005), research also points to continuity in parent–offspring relationships. For example, both social learning theory (Bandura, 1973) and attachment theory (e.g., Ainsworth, 1989; Bowlby, 1969) assume that interaction patterns learned and enacted during childhood and adolescence will continue to manifest themselves in young adults’ relationships. In accordance with these perspectives, parent–offspring relationships during childhood are predictive of parent–offspring relationships during adolescence and adulthood (e.g., Aquilino, 1997; Belsky, Jaffee, Hsieh, & Silva, 2001; Dalton, Frick‐Horbury, & Kitzmann, 2006; Whitbeck, Hoyt, & Huck, 1994). Specifically, more warmth, intimacy, and cohesion during childhood and adolescence have been associated with more emotional closeness, support, and contact with parents during early adulthood (e.g., Aquilino, 1997; Belsky et al., 2001). In contrast, more conflict with parents during adolescence has been associated with more conflict with parents during young adulthood (Aquilino, 1997). Thus, to a certain extent, parental interactions with their underage offspring show continuity in their interactions with their adolescent and adult offspring.

### 1.4 Childhood maltreatment history and autonomic reactivity

Studies that include ANS activity measures may provide insight into the mechanisms underlying the effects of childhood maltreatment experiences on parenting behavior. The ANS is part of the peripheral nervous system that is responsible for regulating the internal organs and glands (Larsen, Schneiderman, & De Carlo Pasin, 1986). The ANS consists of two subsystems: the parasympathetic nervous system (PNS) and the sympathetic nervous system (SNS). Generally, the SNS mobilizes the body's fight‐or‐flight response, whereas the PNS controls homeostasis and the body's rest‐and‐digest response. Thus, stress typically activates the SNS while the PNS is inhibited (Michels et al., 2013; Viamontes & Nemeroff, 2009). A general measure to monitor changes in cardiac SNS activity is the pre‐ejection period (PEP), which is determined by systolic time intervals and reflects cardiac contractility (Newlin & Levenson, 1979). The degree of cardiac control by the PNS is commonly assessed as the amplitude of respiratory sinus arrhythmia (RSA; Porges, 1995). RSA is an indirect measure of vagal tone, which reflects parasympathetic influence on the heart via the vagus nerve, a cranial nerve pertaining to the PNS. Therefore, in a typical response to stress, PEP decreases due to shortening of the systolic period and RSA decreases due to cardiac vagal withdrawal.

Studies that focused on the association between childhood maltreatment and ANS reactivity to psychosocial stressors mainly reveal physiological *hyper‐*reactivity in response to stressors (i.e., overarousal) following childhood maltreatment (e.g., Heim et al., 2000; Dale et al., 2009), but *hypo‐*reactivity (i.e., underarousal) has also been found (e.g., Ginty, Masters, Nelson, Kaye, & Conklin, 2017). In addition, three studies examined the impact of (stress inducing) infant emotional signals on parental ANS reactivity (Buisman et al., 2018; Casanova et al., 1994; Reijman et al., 2014). Casanova et al. (1994) found that mothers with a history of childhood abuse exhibited increases in skin conductance (reflecting increased SNS activity) while viewing a video of a smiling infant, but not while viewing a video of a crying infant. The opposite pattern was observed for mothers without a history of abuse. This may indicate that mothers with a history of abuse are less sensitive to child negative emotional states. Another study on maltreatment and autonomic reactivity revealed that PEP recovery values (i.e., values during a 4‐min baseline of neutral images after exposure to infant cry sounds) decreased in non‐maltreating mothers with few maltreatment experiences, whereas they increased in nonmaltreating mothers with extensive maltreatment experiences (Reijman et al., 2014). For maltreating mothers, PEP recovery did not vary as a function of their experienced maltreatment. The authors suggest that having experienced extensive maltreatment may result in a maladaptive PEP recovery pattern that is similar to that of maltreating mothers. Finally, Buisman et al. (2018) found no evidence for altered ANS reactivity to infant emotional signals following childhood neglect, but did find that childhood neglect experiences were associated with a higher heart rate and a shorter PEP during the entire infant vocalization paradigm, which may indicate chronic cardiovascular *hyper*‐arousal (Brosschot, Pieper, & Thayer, 2005). Contrary to ANS reactivity, which may indicate responsivity to distress, sustained (i.e., basal) ANS activation may more generally represent the capacity for emotion regulation (Appelhans & Luecken, 2006). In conclusion, studies have shown differential ANS regulation and reactivity to psychosocial and attachment‐related stressors following maltreatment. Yet, empirical research on the link between child maltreatment experiences and parents’ ANS reactivity in the context of stressful parent–offspring interactions is lacking. More insight into what happens “under the skin” when parents with a history of childhood maltreatment interact with their offspring may help researchers and practitioners better understand how maladaptive parenting behaviors are transmitted from one generation to the next.

### 1.5 Autonomic reactivity and parenting behavior

Neurobiological frameworks underscore the significance of irregularities in physiological system functioning in shaping individuals’ interactions with significant others and thus provide a foundation for associations with maladaptive parenting behaviors (Bridges, 2008). Research examining parental ANS functioning during stressful parent–offspring contexts provides some empirical support for an association between physiological dysregulation and maladaptive parenting behavior, particularly regarding PNS activity. For example, Lorber and O'Leary (2005) found that mothers who displayed harsh discipline with their preschool children during a task designed to elicit challenging toddler behavior showed corresponding decreases in RSA (i.e., PNS withdrawal). RSA decreases, however, have also been associated with adaptive parenting behavior. For example, a greater degree of maternal PNS withdrawal has been associated with more sensitive (Moore et al., 2009) and less negative intrusive parenting behavior (Mills‐Koonce et al., 2009) during parenting challenges. Studies thus indicate mixed results on the association between autonomic reactivity during parenting and the quality of parenting behavior, and—to our knowledge—this association has not yet been examined using indicators of the SNS. Such research is therefore needed to further our understanding of the psychophysiology of real‐time parenting behaviors.

### 1.6 The current study

In order to gain further insight into the link between childhood maltreatment experiences and subsequent parenting, the present study examined associations between parents’ childhood maltreatment experiences and their autonomic and behavioral reactivity during a parent–offspring conflict interaction task that has been shown to elicit stress responses in previous studies (e.g., Beijersbergen, Bakermans‐Kranenburg, van IJzendoorn, & Juffer, 2008; Eisenberg et al., 2008). We hypothesized that parents who experienced childhood maltreatment (physical & emotional neglect and physical & emotional abuse) would show less warmth and emotional support and more negativity during the parent–offspring conflict interaction task. Furthermore, we hypothesized that parents with childhood maltreatment experiences were more likely to exhibit autonomic *hyper*‐reactivity (PEP and RSA decreases) during the parent–offspring conflict interaction task. We also hypothesized that parental behavior and autonomic reactivity would be interrelated. However, due to mixed findings from previous studies, no specific hypotheses were made regarding the directions of the associations. Furthermore, since research has shown that ANS dysregulation follows childhood maltreatment experiences (e.g., Heim et al., 2000) and may also underlie maladaptive parenting (Bridges, 2008), ANS dysregulation may act as a mechanism in the association between childhood maltreatment experiences and parenting. Although measured concurrently, in case of significant associations between ANS reactivity and parenting, we also explored whether ANS reactivity mediated the association between childhood maltreatment experiences and parenting. Finally, we tested whether childhood maltreatment experiences were associated with basal autonomic activity, and we hypothesized that childhood maltreatment experiences would be associated with overall sustained physiological arousal.

## 2 METHOD

### 2.1 Sample

#### 2.1.1 Recruitment

The current sample was part of a larger sample from the 3G parenting study, a family study on the intergenerational transmission of parenting styles, stress, and emotion regulation (see also Buisman et al., 2018; Compier‐de Block et al., 2016). Participants of the 3G parenting study were recruited from three participant pools: (a) The Netherlands Study of Depression and Anxiety (Penninx et al., 2008); (b) the Longitudinal Internet Studies for the Social Sciences panel (Scherpenzeel, 2011); and (c) a study on parenting (Joosen, Mesman, Bakermans‐Kranenburg, & van IJzendoorn, 2013). From two of these studies, we recruited participants who reported having experienced some form of maltreatment during their childhood, and from the third study, all participants were invited. In order to protect the privacy of our participants, we cannot disclose from which study we recruited maltreated participants. The participants from these three studies served as target participants. In total, 63 participants from different families agreed to take part in the 3G parenting study. After their consent, we invited their family members (parents, partners, children, adult siblings, nephews, nieces, and in‐laws) to participate as well. Family members had to be at least 7.5 years of age to be invited. Families were included if at least two first‐degree relatives from two generations were willing to participate. This resulted in a total of 395 participants from 63 families with two to four generations and an average of 6.27 family members per family (range: 2–23), who agreed to participate in the 3G parenting study.

#### 2.1.2 Participants

Parents with offspring who were at least 7.5 years of age (*n* = 229) were eligible for the conflict interaction task (i.e., revealed differences task, Strodtbeck, 1951) and were included in the current study. Parents whose offspring did not participate, but with data on experienced maltreatment, baseline autonomic activity, and demographic covariates were included in the analyses. Participants (131 women, 98 men) were on average 52.7 years of age (range: 26.6–88.4 years) and were mainly Caucasian (97%). The majority of participants (66%) had an advanced secondary school or vocational school diploma, 25% held a college or university degree, 7% had completed only elementary school or a short track of secondary school, and 2% of the participants did not report their education. Participating offspring (*n* = 306) were on average 24.6 years of age (range: 7.5–65.6). Approximately half of the parents (*n* = 111) completed the interaction task with multiple offspring. For these parents, we selected the data of the interaction task that was done with the offspring whose age was closest to the mean age of the offspring in order to reduce the variance in offspring's age. Out of 229 participants, 30 (13%) participated both as parent and as offspring in the conflict interaction task.

### 2.2 Procedure

Nuclear families were invited for a 7‐hr laboratory visit at the Leiden University Medical Centre. Participants with offspring visited the laboratory twice—once with their family of origin (parents and siblings) and once with their partner and offspring. A laboratory visit involved questionnaires, computer tasks, family interaction tasks, and collection of saliva and hair samples, and during specific tasks, skin conductance and heart rate were measured. Eligible participants were also invited for a functional magnetic resonance imaging session. Informed consent was obtained from all participants. Ethical approval was obtained from the Ethics Committee of the Leiden University Medical Centre. Data collection took place from March 2013 to May 2016.

### 2.3 Measures

#### 2.3.1 Demographic information

Age, gender, and socio‐economic status (SES) were included as background variables. Participants filled out a questionnaire with questions about household income and highest completed education. Yearly household income was measured on a 7‐point scale ranging from (1) less than € 15,000 to (7) more than € 65,000. Education was also rated on a 7‐point scale. Based on standardized household income and standardized completed educational level, a composite SES score was calculated.

#### 2.3.2 Childhood maltreatment

Childhood maltreatment was measured using subscales of the Conflict Tactics Scales: Parent‐child (CTSPC; Straus, Hamby, Finkelhor, Moore, & Runyan, 1998) and the Childhood Trauma Questionnaire (CTQ; Bernstein et al., 1994). Participants completed questions about experienced child maltreatment before the age of 18 years by their father and mother separately. If participating in the study, their parents filled out a version in which they were asked whether they had conducted maltreating behaviors toward their offspring before they had reached the age of 18 years. Parent‐report information on maltreatment was available for only 27% of the participants. We therefore decided to run all analyses with self‐report information on experienced maltreatment in the main analyses. As a check, we conducted sensitivity analyses using a multi‐informant score for which—if available—the information from participants and their parents was combined. Analyses using the multi‐informant score yielded similar parameter estimates for the models.

To match the response categories of the CTS and CTQ, we used a 5‐point scale for both questionnaires ranging from 1 = *never* to 5 = *almost always* for all items. The combined questionnaire consisted of four subscales. *Psychological Aggression*(i.e., emotional abuse; CTSPC) was comprised of five items (e.g., “Shouted, yelled, or screamed at me”). Cronbach's alphas were adequate: *α*_mother_ = 0.81, *α*_father_ = 0.74. *Physical Assault*(i.e., physical abuse; CTSPC) was comprised of 13 items, including corporal punishment (five items, e.g., “Being spanked on the hand, arm or leg with a bare hand”), severe assault (four items, e.g., “Being hit with a fist or kicked hard”), and very severe assault (four items, e.g., “Being burned or scalded”). Cronbach's alphas for *Physical Assault* were excellent: *α*_mother_ = 0.91, *α*_father_ = 0.91. The *Emotional* *Neglect* scale included one item from the CTSPC and five items from the CTQ, which were reverse coded for the purpose of analysis (e.g., “My father/mother never told me he/she loved me”). Cronbach's alphas for *Emotional* *Neglect*were excellent: *α*_mother_ = 0.94, *α*_father_ = 0.92. The *Physical Neglect*scale (CTSPC) consisted of four items (e.g., “My father/mother was not able to make sure I got the food I needed”). Cronbach's alphas were adequate: *α*_mother_ = 0.68, *α*_father_ = 0.61.

Total “abuse” and “neglect” history scores were calculated from the four subscale scores (Emotional and Physical Abuse, and Emotional and Physical Neglect) of parents’ self‐reported experienced maltreatment. Scale scores were made up of the highest score for mother or father (e.g., the highest score of *Emotional Abuse by mother*and *Emotional Abuse by father* was used as score for *Emotional Abuse*). Next, an overall *Abuse*score was created by averaging Emotional and Physical Abuse, and an overall *Neglect*score was created by averaging Emotional and Physical Neglect. Pearson correlations revealed significant correlations between Emotional and Physical Abuse (*r*(229) = 0.71, *p* < 0.001) and Emotional and Physical Neglect (*r*(229) = 0.42, *p* < 0.001).

Because the distribution of the CTS data was skewed to the right, scores were log‐transformed and then multiplied by 10 to scale up the variance.

#### 2.3.3 Parental behavior during a parent–offspring conflict interaction task

Using a parent–offspring conflict interaction task, also known as revealed differences task (Strodtbeck, 1951), we investigated the patterns of interaction between parents and offspring. This conflict interaction task has been used in previous research exploring individual functioning and has proven to be a meaningful index of both parenting behavior as well as physiological arousal and regulation (Beijersbergen et al., 2008; Eisenberg et al., 2008). A dyad, consisting of a parent with one offspring at a time, was asked to discuss and try to reach consensus on an issue on which they disagreed. Participants were each asked to fill out a questionnaire listing common parent–offspring discussion topics (e.g., underage offspring: bedtime, homework; adult offspring: raising grandchildren, time spent with family) and to select five topics that had been discussed most often during the past month. In addition, they were asked to indicate how they felt discussing these topics. Based on this information, a research assistant selected the two topics that participants felt most strongly about, preferably topics that were reported by both parent and offspring as something they often discussed at home. The dyads were then brought to a separate room and were instructed to discuss the topics for 10 min and to try to reach consensus. The interactions were filmed, and there were no other people in the room during the interactions.

The interactions were coded with The Supportive Behavior Task Coding Manual, version 1.1 (Allen et al., 2001). Minor adaptations (clarification and fine‐tuning of constructs) were made to the coding system to fit the current study. Parents and offspring received scores for *Warmth*, *Negativity, and Emotional support*.* Warmth*deals with the extent to which a person demonstrates warmth toward the other, that they care about the other, value, and genuinely like the other. This includes verbal expressions (e.g., verbally empathizing), a warm tone of voice, warm facial expressions, and body postures/behaviors showing warmth and a sense of building the relationship (e.g., touching). The 9‐point rating scale ranged from (1) no signs of warmth (i.e., “You can't tell if the person likes or cares about the other”) to (9) clear signs of warmth (i.e., “The participants’ behavior overall gives a warm feeling to the interaction”). *Negativity*captures the level and persistence of tension, hostility, dissension, or antagonism directed at the conversational partner. Examples of negativity are stonewalling, negative statements of the other, eye rolling, loud sighing, interrupting the other, and negative teasing (sarcasm). Negativity was rated on a 9‐point rating scale ranging from (1) demonstrations of negativity are absent to (9) the person is very negative (i.e., “The negativity endures throughout the discussion and is disruptive to the interaction.”). Negativity scores were skewed to the right and therefore logarithmically transformed. *Emotional support*represents the amount of support that a person gives to the other to indicate an understanding or support of the feelings of the other person, through naming the emotion, recognizing the feelings, sympathizing (e.g., “I'm sorry you feel that way”), or eliciting emotional information. The 9‐point rating scale ranged from (1) absence of emotional support (i.e., “No attempts to emotionally support the other person are made.”) to (9) high emotional support (i.e., “The supporter clearly recognizes the other's emotional distress and makes clear attempts to draw the other out”).

In addition, we coded *Discussion of a problem*and *Supportive role*. During the interaction task, some dyads discussed topics that were not seen as problems. *Discussion of problem*was coded on a dichotomous scale where 0 indicates the absence of discussing a problem and 1 indicates that a problem was discussed. *Supportive role* captures the extent to which a person was expected to be supportive during the conversation. This code was added because conversational partners sometimes do not disclose any emotional information and therefore the other partner is not expected to respond in an emotionally supportive way (i.e., there is no information to respond emotionally supportive to). A score of 1 was assigned when the parent disclosed no emotional information (only the offspring disclosed emotional information) during the conversation and a score of 0 when parents disclosed emotional information.

The interactions were coded by the first author and three trained research assistants. The two participants of the dyad were coded independently. Coders were blind to maltreatment history and maltreatment perpetration of the individual they coded, as well as to the scores of the individual in interaction with other family members. Extensive training in using the coding system was given by the first and last author of this manuscript. After training, 20 videos (40 individuals) were coded to assess reliability. Then, a period of 5 months followed in which videos were coded individually. During biweekly meetings, videos were discussed in order to minimize observer drift. In addition, halfway the coding period another set of 20 videos were coded, which resulted in a total reliability set of 40 videos (80 individuals). Interrater reliability between all pairs of observers was adequate to good, with intraclass correlations for the separate pairs (ICCs; single measures, absolute agreement) ranging from 0.71 to 0.82 for *Warmth*, 0.66 to 0.78 for *Negativity,*and 0.72 to 0.80 for *Emotional Support*. Cohen's Kappa showed perfect interrater reliability (*k* = 1.0) for *Discussion of a problem* and good interrater reliability (*k*_range _= 0.70–0.80) for *Supportive role*.

Each dyad consisted of a parent and an offspring (who could be an adult as well). Because the focus of the current study was on parental physiological and behavioral responses, we decided to only use the scores of the parents in the analyses. Of the 229 parents, interactions of 10 parents were missing because their children did not participate in the study, and interactions of 11 parents were lost because of technical problems with recording the interaction. Moreover, scores of parents were excluded when no problem was discussed (*n = *8).

#### 2.3.4 Autonomic (Re)activity

Before and during the conflict interaction task, electrocardiogram (ECG) signals and impedance cardiogram (ICG) signals were recorded using an ambulatory monitoring system (VU‐AMS5 fs; TD‐FPP, Vrije Universiteit, Amsterdam, the Netherlands). The ECG signal was recorded continuously using three disposable pregelled Ag–AgCl electrodes (ConMed, New York, USA) that were placed below the right collarbone 4 cm to the right of the sternum, 4 cm under the left nipple, and at the lateral right side. For the ICG, four electrodes were attached at the top end of the sternum between the tips of the collarbones, on the spine (at least 3 cm above the previous one), at the low end of the sternum where the ribs meet, and again on the spine (at least 3 cm under the previous one).

A complementary VU‐DAMS software package derived interbeat interval time series (IBIs) by visual peak detection of the R‐wave. We inspected each ECG recording and corrected it manually when necessary (in accordance with VU‐DAMS instructions). From each ICG recording, PEP was scored manually for the baseline task and the interaction task by the first author and a trained research assistant. Interrater reliability (ICC; single measures, absolute agreement) on 40 cases was excellent (ICC = 0.96). The respiration signal was obtained from filtered (0.1–0.4 Hz) thoracic impedance signal. The beginning and end of inspiration and expiration were detected by an automatic scoring algorithm. RSA was derived by the peak‐valley method, in which the respiratory time series and the interbeat intervals (IBI) are combined to calculate the shortest IBI during HR acceleration in the inspiration phase, and the longest IBI during deceleration in the expiration phase (De Geus, Willemsen, Klaver, & Van Doornen, 1995). RSA was defined as the difference between the longest and the shortest IBI. Scoring of the respiration signal and the IBI was done automatically. RSA values were logarithmically transformed because of a skewed distribution.

Prior to the interaction task, participants were presented with a series of neutral pictures and were asked to describe them for 2 min to measure resting autonomic activity. This baseline task was chosen because participants speak during the interaction task and speech has been shown to affect PNS and SNS measures (e.g., Bernardi, Porta, Gabutti, Spicuzza, & Sleight, 2001;Sloan, Korten, & Myers, 1991) due to alterations in the respiratory cycle while speaking and to motor activity in the face and throat. For the evaluation of autonomic reactivity to the interaction task, a residualized change score was computed by regressing PEP and RSA scores during the interaction task on PEP and RSA scores during baseline. The residuals were then used as measures of RSA and PEP reactivity, respectively. Residualized change scores are referred to as “base‐free” measures of change and avoid some of the reliability concerns with difference scores (MacKinnon, 2008). Lower residualized change scores for RSA and PEP indicate greater PNS withdrawal and SNS activation from baseline to task, respectively.

As with the behavioral measures, only parent's physiological responses were examined in the current study. We checked for outliers in the distribution of baseline scores, interaction task scores, and the residualized scores. For RSA, one participant showed an outlying value during baseline, which was winsorized, that is, the difference between the two next highest values was added to the next highest value with standardized value <3.29 (Tabachnick & Fidell, 2001). For PEP, two participants showed outlying values for the residualized change score, which were winsorized as well. Due to technical problems, autonomic activity scores were missing during baseline and the interaction task for eight and 20 participants, respectively. Furthermore, eight participants had missing autonomic activity scores because of heart conditions incompatible with ECG analysis, and an additional 10 participants had no data for PEP because of flat ICGs.

#### 2.3.5 Physical exercise and smoking

Physical condition and smoking may influence heart rate (De Geus, Boomsma, & Snieder, 2003; Hayano et al., 1989). We therefore asked participants whether they had engaged in physical exercise or sports in the previous week, and whether they smoked or not.

### 2.4 Analyses

Pearson correlations between maltreatment, parental interactive behavior, and autonomic (re)activity were computed. Subsequently, we computed a structural model using the package *lavaan* (Rosseel, 2012) in R (R Core Team, 2013) to estimate the associations between experienced maltreatment, parental interactive behavior, and autonomic reactivity during the interaction task. Age, gender, and SES were included as covariates to ensure that demographic information did not confound the results. Supportive role was included as a covariate for emotional support. Moreover, respiration rate was taken into account for RSA, as RSA is susceptible to fluctuations in breathing pattern (Bernardi et al., 2001). Finally, smoking and physical exercise were included in the regression equations for autonomic reactivity and excluded when *p*values exceeded 0.05.

Twelve percent of all values were missing, with missingness rates per variable ranging from 0% to 24%. Little's missing completely at random (MCAR) test was not significant (*χ* ^2^ **= 113.71, *df *= 111, *p *= 0.411) indicating that data was MCAR. Therefore, missingness was handled using full information maximum likelihood (FIML).

Sixty‐nine percent of the parents shared a household together, and in case both parents participated with multiple offspring, the interaction with the same offspring was included in the analyses. To correct for family dependency, robust standard errors were calculated using the package *lavaan.survey.fiml*. This package applies the same method as *lavaan.survey* (Oberski, 2014) but in addition allows for models with missing data estimated using FIML. In *lavaan.survey.fiml,* the structural model parameter estimates are aggregated, that is, parameter estimates are aggregated over clusters and no explicit modeling of the effects of clusters is involved. See Muthen and Satorrah (1995) for details on this procedure.

The covariance matrix was used to estimate the fit of the models. The maximum likelihood estimator was used to estimate the model parameters (Rosseel, 2012). The chi‐square statistic is reported, but assessment of fit was based on the comparative fit index (CFI) and the root mean square error of approximation (RMSEA), since the chi‐square statistic is very sensitive to sample size. Acceptable and excellent model fit is indicated by CFI values >0.90 and 0.95, respectively, and by RMSEA values smaller than 0.08 and 0.06, respectively (Hu & Bentler, 1999).

## 3 RESULTS

### 3.1 Preliminary analyses

Descriptive statistics and correlations of experiences of childhood maltreatment, parental interactive behavior, autonomic (re)activity, and demographics are included in Table 1. Age was significantly negatively correlated with emotional support, negativity, RSA (baseline and reactivity), and significantly positively correlated with PEP during baseline. In addition, SES correlated positively with warmth, emotional support, and baseline RSA. As expected, warmth, negativity, and emotional support were intercorrelated, but correlations were lower than *r* = 0.55, meaning that these represent clearly distinctive parental behaviors. Likewise, abuse and neglect were correlated, though moderately (*r* = 0.53), meaning that they often co‐occur yet represent partially distinct types of maltreatment.

**Table 1:** Mean (*SD*) and Pearson correlations between study variables

	*M* (*SD*)	1	2	3	4	5	6	7	8	9	10	11
1. Age	52.70 (13.19)	‐										
2. Gender (% female)	57	0.03	‐									
3. SES	−0.02 (0.81)	−0.25***	−0.12	‐								
4. Warmth	5.37 (1.85)	−0.12	0.07	0.15*	‐							
5. Negativity	0.28 (0.27)	−0.18*	−0.01	−0.09	−0.55***	‐						
6. Emotional support	3.20 (1.91)	−0.17*	0.08	0.16*	0.54***	−0.30***	‐					
7. RSA baseline	1.60 (0.30)	−0.52***	−0.06	0.24**	0.09	0.12	0.06	‐				
8. PEP baseline	119.58 (26.21)	0.18*	−0.00	−0.07	0.02	−0.07	0.02	−0.06	‐			
9. RSA reactivity	0.00 (1.00)	−0.15*	0.23**	−0.08	0.02	0.18*	0.01	0.00	0.06	‐		
10. PEP reactivity	0.00 (1.00)	−0.03	−0.09	0.04	0.14	−0.05	0.08	0.10	0.01	−0.01	‐	
11. Exp abuse	2.04 (1.31)	0.03	−0.02	0.01	−0.14*	0.19**	−0.10	−0.01	0.02	−0.06	−0.21**	‐
12. Exp neglect	2.88 (1.48)	0.12	0.11	−0.04	−0.05	0.05	−0.04	−0.23**	0.05	−0.18*	−0.26**	0.53***

*Note*. Exp = experienced. Gender: 0 = male, 1 = female. Values for RSA, negativity, experienced abuse and experienced neglect were log‐transformed. RSA and PEP reactivity scores represent residualized change scores.

*p < 0.05. **p < 0.01. ***p < 0.001.

Experienced abuse correlated negatively with warmth and positively with negativity. Furthermore, experienced neglect correlated negatively with RSA reactivity, meaning that parents who experienced higher levels of neglect showed greater PNS reactivity in the form of PNS withdrawal during the task. In addition, there was a significant negative correlation between experienced abuse and neglect and PEP reactivity, meaning that parents with higher levels of maltreatment experiences exhibited a shorter PEP during the task indicative of greater SNS reactivity. Finally, experienced neglect correlated negatively with baseline RSA, meaning that parents who experienced higher levels of neglect had a lower baseline heart rate variability. Since RSA during baseline was not associated with experienced abuse or with parental behavior, and no associations were found between PEP during baseline and the other study variables, we decided not to test a structural model of childhood maltreatment, parental interactive behavior, and baseline autonomic activity.

### 3.2 Structural model

**Figure 1:**
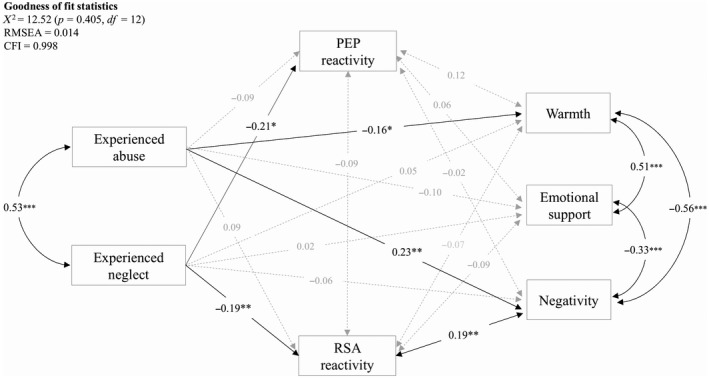
Structural model of childhood maltreatment experiences, parental interactive behavior, and autonomic reactivity during parent–offspring interactions. Respiratory sinus arrhythmia (RSA) reactivity and pre‐ejection period (PEP) reactivity represent residualized change scores. Lower residualized change scores for RSA and PEP reflect greater parasympathetic nervous system withdrawal and sympathetic nervous system activation from baseline to task, respectively. Standard errors are corrected for the dependency in the data. The nuclear family was used as the cluster unit for the correction. Path coefficients are standardized regression weights. Dotted lines indicate nonsignificant paths. Covariates (ages, gender, socio‐economic status, supportive role, respiration rate, and smoking) are not displayed. *p < 0.05; **p < 0.01, ***p < 0.001

As shown in Figure 1, the hypothesized structural model of child maltreatment experiences, parental behavior, and autonomic reactivity exhibited an excellent fit: *X* ^2^(12) = 12.52, *p* = 0.405; CFI = 0.998; RMSEA = 0.014. This indicated that our hypothesized model adequately described the data. The individual pathways are described below.

#### 3.2.1 Experienced neglect

Age (*β *= −0.18, *SE* = 0.01, *p *= 0.005), SES (*β *= −0.17, *SE* = 0.09, *p *= 0.018), and respiration rate (*β *= −0.30, *SE* = 0.06, *p *< 0.001) were significantly negatively associated with RSA reactivity, indicating that older parents, parents with a higher SES, and parents with a higher respiration rate showed greater PNS reactivity (i.e., PNS withdrawal) during interacting with their offspring. In addition, gender was significantly associated with RSA reactivity (*β = *0.24, *SE* = 0.15, *p *= 0.002), such that mothers showed a greater degree of PNS withdrawal while interacting with their offspring. Smoking, but not physical exercise, was significantly negatively associated with RSA reactivity (*β *= −0.22, *SE* = 0.14, *p *< 0.001) and therefore included as an additional covariate in the regression equation for RSA.

Taking demographic variables and smoking into account, experienced neglect showed, as expected, a significant negative association with RSA reactivity (*β *= −0.19, *SE* = 0.05, *p *= 0.005). This indicates that parents who experienced higher levels of neglect during their childhood showed more PNS withdrawal during the interaction task with their own offspring. Furthermore, in accordance with our expectations, experienced neglect was significantly negatively associated with PEP reactivity (*β *= −0.21, *SE* = 0.06, *p *= 0.022), indicating that parents who experienced more childhood neglect exhibited stronger SNS reactivity during the task. Age, gender, and SES (*ps *> 0.295) were taken into account for PEP reactivity to control for demographic characteristics. Smoking and physical exercise were not significantly associated with PEP reactivity during the task (*ps *> 0.154) and consequently were not included as additional covariates in the model. Incongruent with our expectations, experienced neglect was not significantly associated with any type of parental behavior (i.e., warmth, negativity, and emotional support), indicating that a history of childhood neglect was associated with physiological responses, but not with behavioral responses as assessed in the current study.

#### 3.2.2 Experienced abuse

A history of childhood abuse was, as expected, negatively associated with warmth (*β *= −0.16, *SE* = 0.12, *p *= 0.046) and positively associated with negativity (*β = *0.23, *SE* = 0.02, *p *= 0.005), indicating that parents who experienced more abuse during their childhood showed less warmth and more negativity toward their offspring. Contrary to our hypothesis, no significant relation was found between parents’ childhood abuse experiences and parents’ emotional support toward their offspring (*β *= −0.10, *SE* = 0.11, *p *= 0.133). In addition, a history of abuse was not significantly associated with RSA reactivity (*β = *0.09, *SE* = 0.05, *p *= 0.199) or PEP reactivity (*β *= −0.09, *SE* = 0.07, *p *= 0.367). Thus, a history of abuse was associated with parents’ behavioral responses, but not with their autonomic reactivity.

#### 3.2.3 Autonomic reactivity and parental interactive behavior

Our expectation that RSA reactivity would be associated with negativity was in part confirmed, although the direction of the association was opposite to our hypothesis. RSA reactivity was positively associated with negativity (*β = *0.19, *SE* = 0.02, *p *= 0.009), indicating that parents who were more negative toward their offspring showed blunted PNS reactivity. Contrary to our hypotheses, no significant associations were found between RSA reactivity and warmth, *β *= −0.07, *SE* = 0.12, *p *= 0.388, or emotional support, *β *= −0.09, *SE* = 0.12, *p *= 0.203. Finally, no significant associations were found between PEP reactivity and any type of parental interactive behavior (*ps *> 0.102), indicating that parental warmth, negativity, and emotional support did not associate with SNS reactivity.

### 3.3 Exploratory analysis: mediation of RSA reactivity

The structural model (Figure 1) showed that experienced neglect was negatively associated with RSA reactivity, which in turn was positively associated with negative behavior. Hence, we explored whether RSA reactivity mediated the association between experienced neglect and negativity. The indirect effect was tested using a bootstrap estimation approach with 1,000 samples. The results indicated that the indirect effect was not significant, *β* = −0.01, *SE* = 0.003, 95% CI = −0.112, 0.102, indicating no mediation of RSA reactivity in the association between experienced neglect and negativity.

### 3.4 Sensitivity analyses

Results did not alter when the 13% participants who participated both as parent and as offspring in the interaction task were randomly included as either parent or offspring.

Moreover, correlations between baseline and activity scores were high for PEP (*r* = 0.94) and for RSA (*r* = 0.88). To examine the robustness of the results on autonomic reactivity, we therefore conducted sensitivity analyses in which autonomic reactivity scores (residualized change scores) in the structural model were replaced with raw autonomic activity scores during the interaction task. Associations between experienced neglect and RSA activity (*β *= −0.22, *SE* = 0.01, *p *< 0.001) and between RSA activity and negativity (*β = *0.15, *SE* = 0.00, *p *= 0.019) remained significant. However, the association between experienced neglect and PEP activity (*β *= −0.08, *SE* = 1.43, *p *= 0.348) was not significant anymore.

## 4 DISCUSSION

The current study is the first to investigate the role of childhood abuse and neglect in parents’ autonomic *and* behavioral responses during a parent–offspring conflict interaction task. Our findings showed that experiences of childhood neglect were uniquely associated with autonomic *hyper*‐reactivity responses, whereas experiences of childhood abuse were uniquely associated with behavioral responses while discussing conflict. Thus, there may be unique consequences of the two types of childhood maltreatment, above and beyond effects they might have in common. Moreover, behavioral responses were positively associated with RSA reactivity, such that parents who showed more negative behavior toward their offspring exhibited blunted PNS reactivity during the conflict interaction task.

### 4.1 Differential consequences of abuse and neglect

Parents who experienced higher levels of neglect during their childhood showed greater RSA reactivity in the form of RSA reduction (i.e., PNS withdrawal) and greater PEP reactivity in the form of PEP shortening (i.e., SNS activity) while discussing conflict with their offspring, indicative of autonomic *hyper‐*arousal. This was in line with our expectations. We also found that childhood neglect was associated with lower basal RSA, indicating chronic cardiovascular *hype*r‐arousal (Brosschot et al., 2005). Given high correlations between baseline and activity assessments (with potential negative implications for residualized change scores), we conducted sensitivity analyses with the raw activity scores (i.e., activity scores during the conflict interaction task uncontrolled for baseline). These analyses showed similar results for RSA reactivity and activity, but not for PEP reactivity and activity. Associations between experienced neglect and RSA thus seem more robust than estimates for PEP. Unexpectedly, no associations were observed between parents’ experiences of childhood neglect and their expressions of warmth, negativity, and emotional support toward their offspring.

Results regarding abuse differed from those of neglect. As expected, and in line with previous research (Vaillancourt et al., 2017), we found that parents who experienced more abuse during their childhood showed less warmth and more negativity while discussing conflict with their offspring. Unexpectedly, experiences of childhood abuse were not significantly associated with RSA or PEP reactivity, indicating that a history of abuse only associated with behavioral responses, but not with autonomic responses.

The present findings suggest that abuse and neglect may differentially affect physiological and behavioral systems. Neglect, which is characterized by unresponsive parenting, may more strongly affect physiological stress responses, whereas abuse, which is characterized by physical or verbal aggressive parenting, may more strongly impact behavioral responses. Child neglect is more likely to reoccur and more intractable to intervention than other forms of child maltreatment—as measured by the percentage of recurrences of child maltreatment following a report to Child Protective Services (Jonson‐Reid, Drake, Chung, & Way, 2003; Lipien & Forthofer, 2004), reduced reunification rates of children in care (i.e., Biehal, Sinclair, & Wade, 2015), and effectiveness of home visitation on recurrences of child maltreatment (i.e., MacMillan et al., 2005). Moreover, neglect can be hard to identify for professionals and therefore may often go unnoticed (Jones & Gupta, 1998). Consequently, child neglect is frequently thought of as a chronic pervasive problem that is difficult to resolve (Hildyard & Wolfe, 2002; Jones & Gupta, 1998). As such, neglect is considered a pervasive chronic stressor, which might fail to buffer individuals from stressors and chronic activation of the ANS system (De Bellis, 2005). When the stress system is exposed to chronic stress, such as neglect, physiological responses may become dysregulated, a condition that has been termed "allostatic load'' (Danese & McEwen, 2012). Indeed, foster children and adopted postinstitutionalized children, who have typically experienced chronic neglect, were found to exhibit elevated ANS reactivity to stressors (Gunnar, Frenn, Wewerka, & Ryzin, 2009; Oosterman, Schipper, Fisher, Dozier, & Schuengel, 2010). Furthermore, in one of our previous studies we found that childhood neglect, but not childhood abuse, was associated with elevated ANS activity of parents in a pseudo parenting context (Buisman et al., 2018). Results of the present study, however, are inconsistent with one other study in which abuse (physical and sexual) was found to be associated with elevated ANS reactivity (Heim et al., 2000). Yet, the researchers did not statistically control for neglect, making it unclear to what extent neglect may have accounted for this association. Moreover, although neglect frequently involves ongoing situations (i.e., Hildyard & Wolfe, 2002; Lipien & Forthofer, 2004), it is not unlikely for abuse to also follow a chronic pattern. To date, there have been no other studies that explicitly compared the effects of abuse and neglect on ANS reactivity and regulation. Additional research that also takes maltreatment chronicity into account is needed to confirm and understand differential consequences of abuse and neglect on ANS functioning of parents, specifically while they are interacting with their offspring.

Childhood abuse, contrary to childhood neglect, may more strongly affect the behavioral system through learning of maladaptive behavioral patterns. Social learning theory (Bandura, 1973) postulates that childhood abuse may result in interpersonal aggression, as children generalize from their experiences with abusers and assume aggression—which can be verbal or physical—to be a suitable and effective form of interpersonal behavior. Such an aggressive interpersonal style may be applied to social interactions in general or may indicate a specific set of parenting behaviors or beliefs that is transmitted from generation to generation. In accordance with this theory, research has consistently found that harsh and abusive parenting is uniquely associated (i.e., controlled for neglect) with more aggression in the offspring (e.g., Cohen, Brown, & Smaile, 2001; Litrownik et al., 2005). Likewise, in the parent–offspring context, research has shown that parents with a childhood history of abuse demonstrate more hostility (e.g., expressed impatience, frustration) toward their children (Pasalich et al., 2016; Vaillancourt et al., 2017). Again, it should be noted that most studies that observed parenting behavior did not statistically control for other types of maltreatment, leaving open the possibility that co‐occurring experiences of neglect accounted for these associations.

The present findings may also indicate that parents with childhood experiences of abuse or neglect are more likely to engage in that same type of maltreating behavior toward their own offspring: That is, neglected parents might be more likely to neglect—but not to abuse—their offspring, and vice versa. Our findings demonstrate that parents with more childhood abuse experiences showed less warm and more negative behavior toward their offspring. This has been more often observed in abusive parents (Borrego, Timmer, Urquiza, & Follette, 2004; Bousha & Twentyman, 1984; Herrenkohll, Herrenkohll, Toedter, & Yanushefsi, 1984). Neglectful parents, on the other hand, are typically characterized by a lack of involvement (Bousha & Twentyman, 1984), which may be unobservable during a task where they are “forced” to interact with their offspring. Instead, these parents may experience more stress as a consequence, which may—in part—explain their enhanced ANS response.

### 4.2 Associations between behavioral and autonomic reactivity

We also found that parents who exhibited blunted PNS reactivity showed more negativity while discussing conflict with their offspring. This was unexpected, because greater PNS reactivity (i.e., PNS withdrawal) is thought to accompany negative emotional states (Beauchaine, 2001). Likewise, in the parent–offspring context, it has been shown that PNS withdrawal is associated with more negative interactions, that is, harsh discipline techniques (Lorber & O'Leary, 2005). However, PNS withdrawal has also been associated with less negative behavior (i.e., intrusive behavior) in mothers participating in a stressful parent–offspring paradigm, but only among mothers with high cortisol levels (Mills‐Koonce et al., 2009). The authors speculated that vagal withdrawal during a parenting challenge may protect mothers from demonstrating negative parenting behavior when they experience either chronic or acute stress. Since we only used ANS activity as an index of physiological reactivity and did not measure cortisol, we cannot test this hypothesis. Replication of our findings including measures of multiple physiological systems is needed to further disentangle associations between these biological systems and complex parenting behaviors.

### 4.3 Limitations and recommendations for future studies

Several methodological issues of our study deserve attention. First, we acknowledge the potential limitations of using a self‐report methodology to investigate child maltreatment. Participants may have difficulty recalling childhood events (Edwards et al., 2001) or may be reluctant to disclose certain experiences or behaviors. Nonetheless, official records may seriously underestimate the prevalence of child maltreatment (e.g., Swahn et al., 2006). Future research should consider using multiple data sources to estimate child maltreatment.

Second, the retrospective assessment of maltreatment precludes conclusions about causality. However, apart from intervention studies with a prospective design, it is ethically impossible to conduct true experiments to examine the consequences of child maltreatment.

Third, our study included parents and offspring with a large age range, while the effects of maltreatment on parent–offspring interactions may differ across different stages of child development. Limited power prevented investigating age differences reliably. Although the large age range increases the generalizability of our findings to parent–offspring relationships across the life span, studies with more power to test group differences or studies with a prospective design may provide insights into the differential impact of childhood maltreatment experiences on parent–offspring interactions across the life span. Because conflict with parents is thought to increase during adolescence (e.g., McGue et al., 2005) and to decrease during adulthood (e.g., Buhl, 2007), future studies may examine whether parents’ history of childhood maltreatment has a more pronounced effect on parent–adolescent interactions compared to parent–child and parent–adult interactions.

Fourth, the present study focused on parental physiological and behavioral responses. However, parent–offspring interactions are not only influenced by parental behavior but reflect a dyadic process, assuming bidirectionality in parent–offspring interactions (Kuczynski, 2003). A direction for future research may be to examine if, for instance, moment‐to‐moment synchrony in the interaction moderates the associations found in the current study.

Furthermore, observations are considered to carry higher ecological validity than questionnaires in examining parenting behavior. Nonetheless, observations for the present study were done in a laboratory setting, which may have affected parents’ behavior due to social desirable responding. Future studies should consider using longer observation periods and home observations, which may elicit typical parenting behavior—including the absence of interactions—to a greater extent.

Moreover, structural equation modeling is considered a strong data analytic technique, and post hoc modifications to establish good model fit were not necessary. Nevertheless, our results may be subject to sampling effects and require replication in different populations and different settings, especially since our sample consisted mainly of Caucasian parents.

Finally, we found no evidence for ANS reactivity as a mediator of the association between childhood maltreatment experiences and parenting. However, ANS reactivity and parenting were measured concurrently. Future studies with a longitudinal design should investigate this mediating process further.

## 5 CONCLUSION

In sum, the present study suggests that behavioral and physiological systems respond differentially to childhood abuse and neglect. While discussing conflict with their offspring, parents who experienced higher levels of childhood abuse responded more strongly on a behavioral level but not on an autonomic level, whereas the opposite pattern was observed for parents who experienced more childhood neglect. Both response patterns, however, may indicate maladaptive emotion regulation and responding that, in turn, may contribute to the transmission of dysfunctional caregiving (Jaffee, 2017). These results support previous suggestions that individuals with maltreatment experiences may benefit from interventions aimed at physiological and behavioral‐based stress regulation (e.g., Maughan & Cicchetti, 2002). Moreover, although different types of maltreatment often co‐occur (Herrenkohl & Herrenkohl, 2009), findings of the present study suggest that they do represent different experiences, highlighting the need to measure different types of maltreatment and to compare their separate influences. This will promote effective targeting of intervention and preventive efforts and understanding of the developmental pathways from unique experiences to adaptive parenthood.

## CONFLICTS OF INTEREST

The authors have no conflicts of interest.
